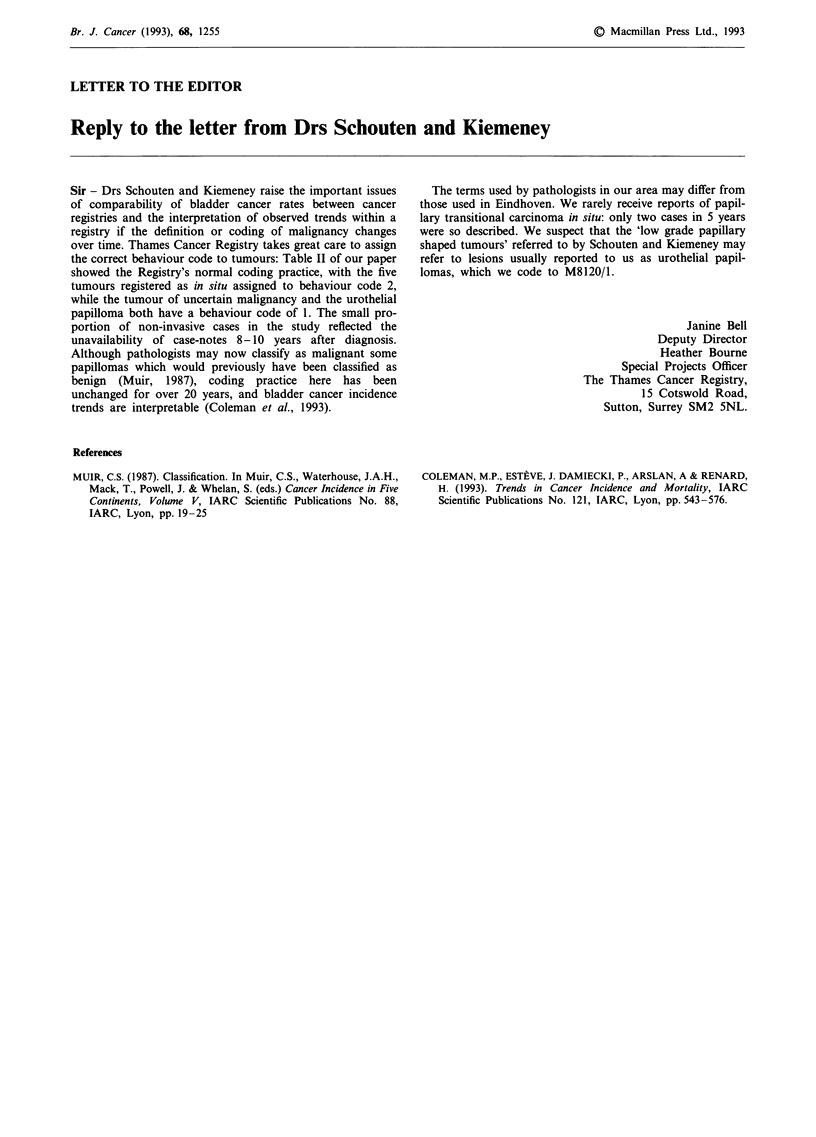# Reply to the letter from Drs Schouten and Kiemeney

**Published:** 1993-12

**Authors:** Janine Bell


*Br. J. Cancer* (1993), **68**, 1255 © Macmillan Press Ltd., 1993

LETTER TO THE EDITOR

### Reply to the letter from Drs Schouten and Kiemeney

**Sir** – Drs Schouten and Kiemeney raise the important issues of comparability of bladder cancer rates between cancer registries and the interpretation of observed trends within a registry if the definition or coding of malignancy changes over time. Thames Cancer Registry takes great care to assign the correct behaviour code to tumours: Table II of our paper showed the Registry's normal coding practice, with the five tumours registered as *in situ* assigned to behaviour code 2, while the tumour of uncertain malignancy and the urothelial papilloma both have a behaviour code of 1. The small proportion of non-invasive cases in the study reflected the unavailability of case-notes 8–10 years after diagnosis. Although pathologists may now classify as malignant some papillomas which would previously have been classified as benign (Muir, 1987), coding practice here has been unchanged for over 20 years, and bladder cancer incidence trends are interpretable (Coleman *et al.*, 1993).

The terms used by pathologists in our area may differ from those used in Eindhoven. We rarely receive reports of papillary transitional carcinoma *in situ*: only two cases in 5 years were so described. We suspect that the 'low grade papillary shaped tumours' referred to by Schouten and Kiemeney may refer to lesions usually reported to us as urothelial papillomas, which we code to M8120/1.

Janine Bell
Deputy Director
Heather Bourne
Special Projects Officer
The Thames Cancer Registry,
15 Cotswold Road,
Sutton, Surrey SM2 5NL.

### References

MUIR, C.S. (1987). Classification. In Muir, C.S., Waterhouse, J.A.H., Mack, T., Powell, J. & Whelan, S. (eds.) *Cancer Incidence in Five Continents, Volume V*, IARC Scientific Publications No. 88, IARC, Lyon, pp. 19–25

COLEMAN, M.P., ESTÈVE, J. DAMIECKI, P., ARSLAN, A & RENARD, H. (1993). *Trends in Cancer Incidence and Mortality*, IARC Scientific Publications No. 121, IARC, Lyon, pp. 543–576.